# External validation of a four-tiered grading system for chromophobe renal cell carcinoma

**DOI:** 10.1007/s10238-024-01314-2

**Published:** 2024-03-30

**Authors:** Shuai Liu, Mingyu Hou, Yu Yao, Jingchang Mei, Lijiang Sun, Guiming Zhang

**Affiliations:** 1 Department of Urology, The Affiliated Hospital of Qingdao University, No. 16, Jiangsu Rd, Qingdao, 266003 China; 2 Department of Pathology, The Affiliated Hospital of Qingdao University, Qingdao, China

**Keywords:** Chromophobe renal cell carcinoma, Grade, Prognosis, Necrosis, Sarcomatoid differentiation

## Abstract

This study aimed to validate the prognostic value of a four-tiered grading system recently proposed by Avulova et al. and to explore the prognostic ability of another four-tiered classification grading system in which there is a separate Grade 3 for tumor necrosis. Grading of chromophobe renal cell carcinoma (ChRCC) by the Fuhrman system is not feasible because of the inherent nuclear atypia in ChRCC. We collected relevant data of 263 patients with ChRCC who had undergone surgery in our hospital from 2008 to 2020. The Kaplan–Meier method was used to calculate the survival rate and Cox proportional hazard regression models to assess associations with cancer-specific survival and distant metastasis-free survival by hazard ratios (HRs) and 95% confidence intervals (CIs). Ten patients died from ChRCC, and 12 developed metastases. The 5 year CSS rates were 95.9%. Grades 2 (HR = 10.9; CI 1.11–106.4; *P* = 0.04), 3 (HR = 33.6, CI 3.32–339.1; *P* = 0.003), and 4 (HR = 417.4, CI 35.0–4976.2; *P* < 0.001) in a four-tiered grading system were significantly associated with CSS in a multivariate setting. However, the difference in CSS between Grades 2 and 3 was not significant (HR = 2.14, 95% CI 0.43–10.63; *P* = 0.35). The HRs of the associations between an exploratory grading system that includes a separate Grade 3 for tumor necrosis and CSS were as follows: Grade 2, 10.2 (CI 1.06–97.9, *P* = 0.045); Grade 3, 11.4 (CI 1.18–109.6, *P* = 0.04); and Grade 4, 267.9 (CI 27.6–2603.3, *P* < 0.001). Similarly, Grades 2 and 3 did not differ significantly. The four-tiered grading system studied is useful for predicting death from ChRCC and metastasis. However, Grade 3 did not more accurately predict risk of death and metastasis than did Grade 2. This was also true for the novel exploratory grading system that classifies tumors with necrosis into a separate Grade 3.

**Supplementary Information:**

The online version contains supplementary material available at 10.1007/s10238-024-01314-2.

## Introduction

Kidney cancer is the third most common tumor of the urinary system, with approximately 431,288 new cases worldwide in 2020, 62.9% of which were in men [1]. Chromophobe renal cell carcinoma (ChRCC) is the third most common pathological type of renal cell carcinoma (RCC), accounting for approximately 5–7% of cases [2, 3]. Most ChRCCs have a favorable prognosis; however, a small percentage of tumors recur quickly and have poor outcomes [4]. Studies have shown that advanced T stage, coagulative necrosis, and sarcomatoid differentiation are associated with more aggressive tumors and poor prognosis [5–9]. Unfortunately, unlike with other tumors for which pathological grade can be used as a prognostic indicator, there is still no accepted pathologic grading system for chromophobe RCC to assist clinical decision-making [10, 11]. The Fuhrman grading system is the most commonly used pathological grading system for renal cell carcinoma (RCC); however, studies have shown that it is not applicable to ChRCC [12–14].

In recent years, although many pathological grading systems have been proposed, none of them has been validated by large multicenter studies [15–18]. One of them, a four-tiered grading system proposed by Avulova et al. [15], was established by adding coagulative tumor necrosis to a three-tiered grading system created by Paner et al. [19]. In this classification, Grade 1 is characterized by an absence of nuclear crowding and atypical cells. Grades 2 and 3 are differentiated by the absence or presence, respectively, of coagulative tumor necrosis. The presence of sarcomatoid differentiation or frank anaplasia is classified as Grade 4. Avulova et al. [15] reported that this four-tiered grading system is an independent predictor of metastasis and cancer-specific death and that its predictive power is stronger than that of the original three-tiered grading system.

The purpose of our study was to investigate the prognostic value of the four-tiered classification grading system by Avulova et al. [15]. Furthermore, we aimed to explore the prognostic value of another novel four-tiered grading system that classifies tumors with necrosis as a separate Grade 3.

## Methods

### Study population

Two hundred and seventy consecutive patients with non-metastatic ChRCC who had undergone surgery at the Department of Urology, the Affiliated Hospital of Qingdao University, from December 2008 to March 2020, were enrolled in this study. Seven patients were excluded for the following reasons: mixed type with components of both clear cell renal cell carcinoma (ccRCC) and ChRCC (*n* = 5), and missing pathological data (*n* = 2). Eventually, 263 patients were included in the study. The Medical Ethics Committee of the Affiliated Hospital of Qingdao University has approved the project protocol. All patients included in this study signed informed consent to treatment. All methods were performed in accordance with relevant guidelines and regulations, such as the Declaration of Helsinki.

### Variables evaluation

Studied patient characteristics included age, gender, body mass index, history of smoking, drinking, hypertension, and diabetes. In addition, studied pathological data included tumor size, pathological T stage (pT), pathological N stage (pN), lymphovascular invasion (LVI), coagulative tumor necrosis, sarcomatoid differentiation, three-tiered grade as proposed by Paner et al. [19], and four-tiered grade as proposed by Avulova et al. [15]. Tumors were staged in accordance with the latest American Joint Committee on Cancer (AJCC) TNM staging system. LVI denotes identification of cancer cells in endothelium-lined space (lymphatics or blood vessels) [20]. Pathological information was based on reviews of the microscopic findings by two urologic pathologists who were blinded to patients' survival outcomes.

### Follow-up

Patients were followed up after nephrectomy every 6 months for 3 years, then annually for 4–5 years, and every 2 years after 5 years. Computed tomography and/or magnetic resonance imaging were used to examine recurrence or metastasis. Outcomes were death from ChRCC and distant metastasis, that is, metastases in locations other than regional lymph nodes. Death was confirmed by death certificates, the final outcomes being adjudicated by a clinician. Duration of follow-up was recorded from the time of surgery to death, distant metastasis, or the end of follow-up.

### Pathological parameters

**Fig. 1:**
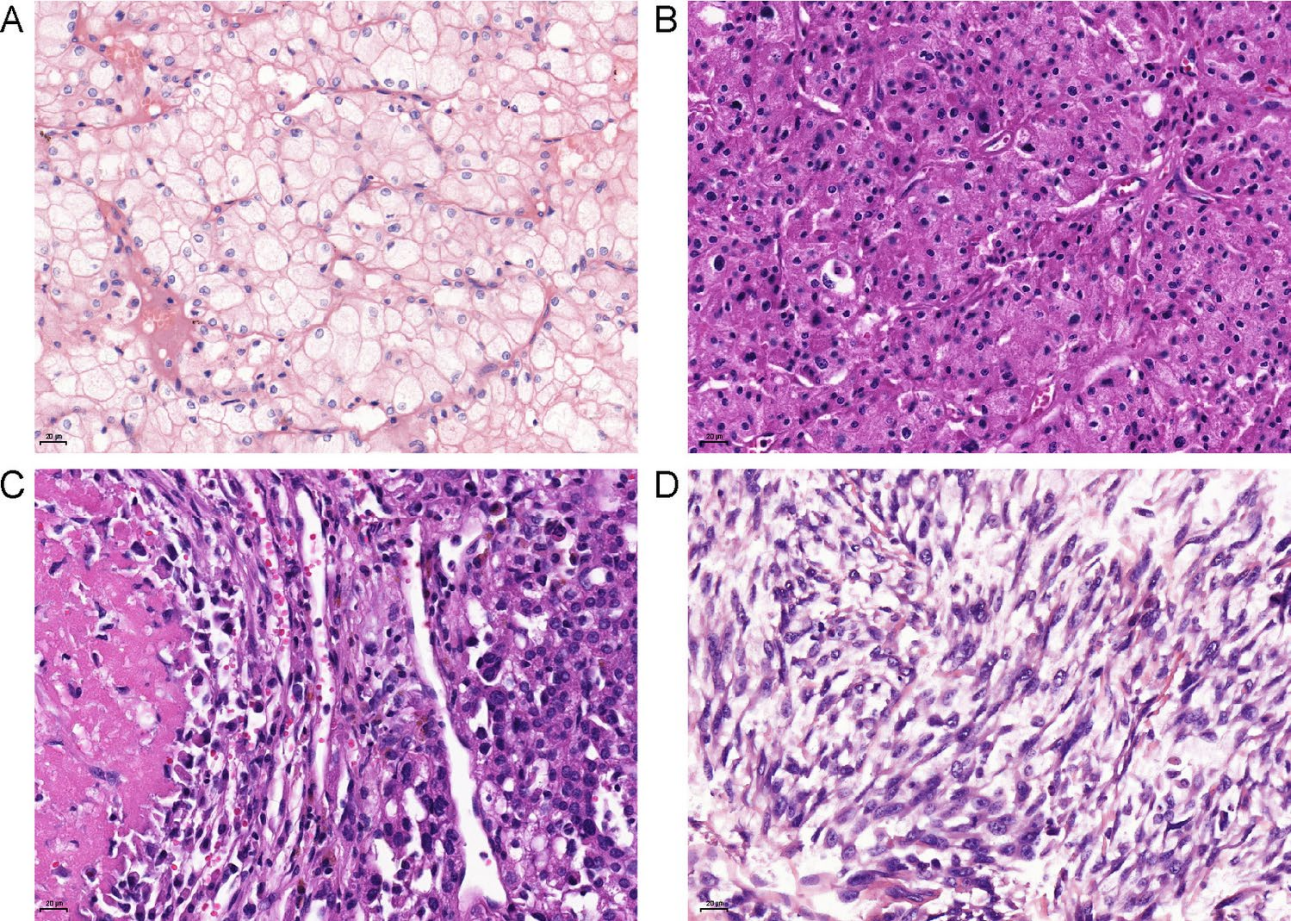
Four-tiered grading system by Avulova et al. Grade 1 (**A**), Grade 2 (**B**), Grade 3 (**B**), and Grade 4 (**D**)

The details of the four-tiered grading system are as follows [15] (Fig. 1): Grade 1: wide internuclear spacing, no nuclear crowding or diffuse atypia, and presence or absence of coagulative tumor necrosis (uniform sheets of dying and degraded tumor cells aggregated into amorphous clots) [21]; Grade 2: Nuclear crowding (cell aggregation characterized by a high spatial nuclear/cytoplasmic density and contact between nuclei observable at × 10 objective) and atypical cells (at least threefold differences in size and markedly irregular nuclear chromatin) without coagulative necrosis; Grade 3: as for Grade 2 but with coagulative necrosis; and Grade 4: sarcomatoid differentiation [22, 23] or frank anaplasia (polylobed nuclei and tumor giant cells).

### Statistical analysis

Differences in patients' characteristics between grades were compared by Kruskal–Wallis tests, Chi-square tests, Fisher’s exact tests, and Spearman rank correlation coefficients. Survival rates were calculated by the Kaplan–Meier method. Cox proportional hazard regression models were used for univariate and multivariate analysis. Associations between pathological grade and survival were analyzed by calculating hazard ratios (HRs) and 95% confidence intervals (CIs). R software 3.6.2 and SPSS version 26.0 were used to perform statistical analysis. *P* values are all two-sided, and *P* < 0.05 was taken to denote statistical significance.

## Results

The study cohort comprised 263 patients with ChRCC, 192, 44, 23, and 4 of whom had Grades 1, 2, 3, and 4 tumors proposed by Avulova et al. [15]. Baseline characteristics according to these four grades are shown in Table 1. The median age was 53.0 years (interquartile range [IQR], 44.0–61.0 years). The patients consisted of 110 men (41.8%) and 153 women (58.2%). The median follow-up time was 4.9 years (IQR, 2.8–7.5 years). There were no significant differences in age, sex, body mass index, smoking or drinking status, history of hypertension or diabetes, Charlson score, and ECOG score between the groups. We present the pathological features corresponding to the four grades in Fig. 1.

**Table 1:** Patients characteristics by ChRCC grade

Feature	Grade 1 (*N* = 192)	Grade 2 (*N* = 44)	Grade 3 (*N* = 23)	Grade 4 (*N* = 4)	*P* value^a^	*P* value^b^
Age (yrs)	53 (44–61)	54 (45–66)	50 (41–56)	59 (56–63)	0.12	0.17
Male	106 (55)	28 (64)	16 (70)	2 (50)	0.4	0.3
BMI (kg/m^2^)	24.8 (22.8–27.1)	24.1 (22.8–26.8)	26.3 (22.4–28.3)	23.5 (22.1–25.5)	0.4	0.4
Symptoms	31 (16)	11 (25)	12 (52)	1 (25)	0.001	< 0.001
Hypertension	46 (24)	15 (34)	6 (26)	0 (0)	0.4	0.4
Diabetes mellitus	20 (10)	3 (7)	1 (4)	1 (25)	0.5	0.7
Charlson score	0 (0–0)	0 (0–0)	0 (0–0)	0 (0–1)	0.8	0.8
Smoking	39 (20)	8 (18)	5 (22)	1 (25)	0.9	0.9
Alcohol	29 (15)	8 (18)	4 (17)	0 (0)	0.9	0.6
Partial nephrectomy	71 (37)	13 (30)	2 (9)	0 (0)	0.02	0.02
Size (cm)	5.0 (3.0–7.0)	5.0 (4.0–6.9)	7.0 (6.0–9.0)	11.5 (10.3–15.6)	< 0.001	0.002
*pT stage*						
pT1	144 (75)	32 (73)	11 (48)	0 (0)	< 0.001	0.01
pT2	34 (18)	3 (18)	7 (30)	2 (50)		
pT3	13 (7)	4 (9)	5 (22)	0 (0)		
pT4	1(1)	0 (0)	0 (0)	2 (50)		
pN1	0 (0)	0 (0)	2 (9)	1 (25)	< 0.001	0.008
LVI	1 (1)	0 (0)	1 (4)	1 (25)	0.02	0.2
Coagulative tumor necrosis	27 (14)	0 (0)	23 (100)	4 (100)	< 0.001	< 0.001
Sarcomatoid differentiation	0 (0)	0 (0)	0 (0)	4 (100)	< 0.001	NA

*BMI* body mass index, *ECOG* eastern cooperative oncology group; and *NA* not applicable. Results are shown as median (interquartile range [IQR]) and frequency (%). *P* values were calculated using Kruskal–Wallis tests for continuous, Chi-square tests and Fisher’s exact tests for unordered categorical variables, and Spearman rank correlation coefficients for ordinal categorical variables

^a^*P* value for comparison of Grade 1 versus 2 versus 3 versus 4. ^b^*P* value for comparison of Grade 1 versus 2 versus 3

**Fig. 2:**
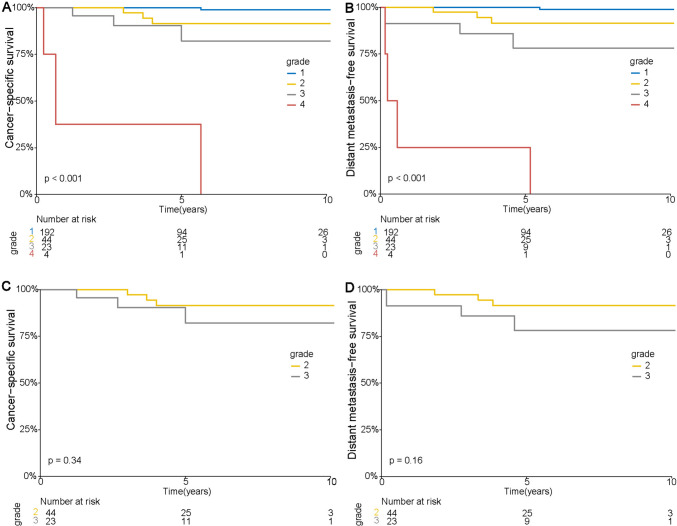
Kaplan–Meier curves of cancer-specific survival (**A**) and distant metastasis-free survival (**B**) stratified by four-tiered classification grading system by Avulova et al.; associations of grades 2 and 3 in four-tiered classification grading system by Avulova et al. with cancer-specific survival (**C**) and distant metastasis-free survival (**D**)

During follow-up, ten patients died from ChRCC, and two died from other causes (hypertension and complications of surgery, respectively). The median follow-up time for living patients was 59.5 months (IQR, 33.0–91.0 months). The 5- and 10-year cancer-specific survival (CSS) rates were 95.9% and 94.2%, respectively. We found significant differences between Grades 1, 2, 3, and 4 in 5-year CSS (100.0% vs. 91.4% vs. 82.1% vs. 37.5%, respectively; Logrank, *P* < 0.001) and 10-year CSS (98.8% vs. 91.4% vs. 82.1% vs. 0.0%, respectively; Logrank, *P* < 0.001) rates (Fig. 2a). Twelve of the 263 patients had distant metastases. The median follow-up without metastases was 4.9 years (IQR, 2.7–7.8 years). The 5- and 10-year distant metastasis-free survival (DMFS) rates for all patients were 95.2% and 93.6%, respectively. The 5-year DMFS rates for patients with Grades 1, 2, 3, and 4 tumors were 100.0% versus 91.6% versus 77.3% versus 25.0%, respectively (Logrank, *p* < 0.001; Fig. 2b).

**Fig. 3:**
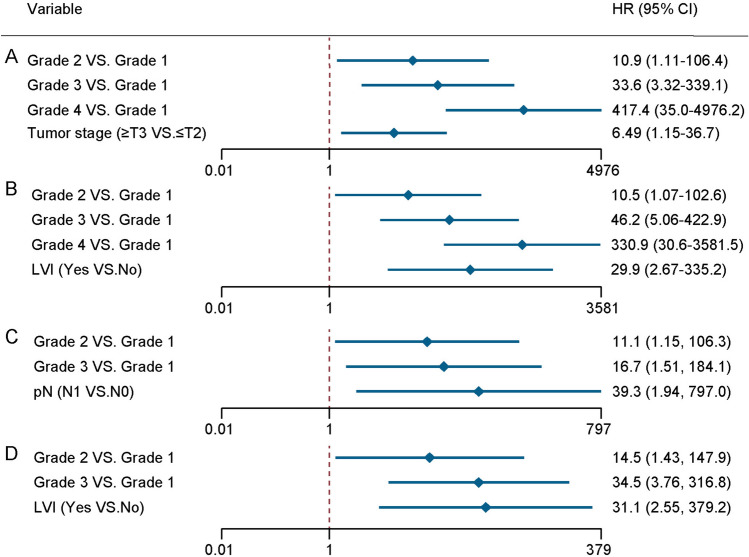
Positive outcomes of multivariable Cox regression models of all patients for CSS (**A**) and DMFS (**B**); positive outcomes of multivariable analyses of non-sarcomatoid ChRCC patients for CSS (**C**) and DMFS (**D**)

Univariate analysis showed that grade in the four-tiered system, tumor size, pT stage, regional lymph node metastasis, LVI, coagulative tumor necrosis, and sarcomatoid change were significantly associated with death from cancer and distant metastasis; all these results are displayed in Table 2. Multivariate analysis revealed that grade in the four-tiered system and pT stage were independent predictors of CSS (Fig. 3a and Table 2). Grade in the four-tiered system and LVI were predictors of DMFS (Fig. 3b and Table 2). Because of the small number of events, we adjusted for patients' characteristics one at a time to minimize the risk of over-fitting. We found that the associations of grade in the four-tiered system with CSS and DMFS were all statistically significant after being adjusted for each characteristic (Table S2).

**Table 2:** Univariable Cox regression analyses of all patients for cancer-specific survival and distant metastasis-free survival

Feature	CSS		DMFS	
	Univariable		Univariable	
	HR (95% CI)	*P*	HR (95% CI)	*P*
Age (per 10 year increase)	1.63 (0.95–2.79)	0.08	1.69 (1.03–2.77)	0.04
Gender (male vs. female)	1.59 (0.46–5.50)	0.5	1.28 (0.39–4.21)	0.7
Symptom (yes vs. no)	1.34 (0.35–5.20)	0.7	1.10 (0.30–4.05)	0.9
Hypertension (yes vs. no)	1.35 (0.35–5.24)	0.7	1.15 (0.31–4.36)	0.8
DM (yes vs. no)	2.02 (0.43–9.54)	0.4	1.87 (0.40–8.69)	0.4
Charlson score (per score increase)	2.06 (0.99–4.30)	0.05	1.79 (0.87–3.70)	0.1
Smoking (yes vs. no)	1.60 (0.41–6.20)	0.5	1.44 (0.38–5.42)	0.6
Alcohol (yes vs. no)	1.37 (0.29–6.48)	0.7	1.23 (0.27–5.71)	0.8
BMI (per 5 kg/m^2^ increase)	0.90 (0.34–2.39)	0.8	1.00 (0.42–2.41)	1.0
Surgery (RN vs. PN)	3.42 (0.43–27.1)	0.2	4.12 (0.53–32.3)	0.18
Tumor size (> 7 vs. ≤ 7 cm)	4.04 (1.1–14.3)	0.03	5.54 (1.67–18.4)	0.005
pT stage (≥ T3 vs. ≤ T2)	5.95 (1.52–23.3)	0.01	6.24 (1.86–20.9)	0.003
pN stage (N1 vs. N0)	117.3 (16.0–857.6)	< 0.001	67.4 (11.2–406.7)	< 0.001
LVI (yes vs. no)	14.0 (1.76–110.8)	0.01	27.7 (5.97–128.2)	< 0.001
Necrosis (yes vs. no)	7.08 (1.99–25.2)	0.003	9.28 (2.78–31.0)	< 0.001
Sarcomatoid differentiation (yes vs. no)	61.8 (15.8–242.1)	< 0.001	57.1 (14.5–225.4)	< 0.001
*Four-tiered ChRCC grade by Avulova et al.*				
1	1.0 (reference)		1.0 (reference)	
2	11.6 (1.20–111.3)	0.03	11.8 (1.23–113.9)	0.03
3	24.9 (2.59–239.8)	0.005	35.0 (3.91–312.8)	0.001
4	302.9 (34.6–3250.8)	< 0.001	379.8 (41.2–3501.9)	< 0.001
*Three-tiered ChRCC grade by Paner et al.*				
1	1.0 (reference)		1.0 (reference)	
2	15.7 (1.90–130.9)	0.011	19.0 (2.34–154.9)	0.006
3	303.1 (31.2–2949.0)	< 0.001	377.2 (40.9–3476.3)	< 0.001
*Exploratory four-tiered ChRCC grade*				
1	1.0 (reference)		1.0 (reference)	
2	10.2 (1.06–97.9)	0.045	10.5 (1.09–100.5)	0.04
3	11.4 (1.18–109.6)	0.04	15.5 (1.73–138.8)	0.01
4	267.9 (27.6–2603.3)	< 0.001	340.8 (36.8–3156.5)	< 0.001

*CSS* cancer-specific survival, *DMFS* distant metastasis-free survival, *DM* diabetes mellitus, *BMI* body mass index, *RN* radical nephrectomy, *PN* partial nephrectomy, *LVI* lymphovascular invasion *HR* hazard ratio, *ChRCC* chromophobe renal cell carcinoma

The results of univariate Cox regression analyses of non-sarcomatoid ChRCC for CSS and DMFS are summarized in Table S1. According to multivariable analysis, higher grade in the four-tiered system and pN1 stage predicted death from cancer in patients with non-sarcomatoid ChRCC (Fig. 3c). Grades 2 and 3 in the four-tiered grading system and LVI were associated with increased risk of metastasis (Fig. 3d).

Although the increased risk of death and metastasis was greater for Grade 3 versus Grade 1 than for Grade 2 versus Grade 1, the difference was small. Univariate analysis showed no significant association between tumor necrosis in patients with Grade 2 or 3 disease in the four-tiered system and poor CSS (HR = 2.14, 95% CI 0.43–10.6; *P* = 0.4) or more numerous metastases (HR = 2.82, 95% CI 0.63–12.6; *P* = 0.18). Separating Grades 2 and 3 did not enhance risk stratification for survival or metastasis (Fig. 2c and 2d). We then investigated the prognostic value of the three-tiered classification grading system of Paner et al*.* [19] (Table S1). One hundred and ninety-two, 67, and 4 patients were identified with Grades 1, 2, and 3 tumors proposed by Paner et al. [19], respectively. The 5 year CSS and DMFS rates for patients with Grades 1, 2, and 3 tumors were 100.0% versus 88.4% versus 37.5% and 100.0% versus 87.0% versus 25.0%, respectively (Logrank, *p* < 0.001; Fig. S1). Univariate analysis showed that grade in the three-tiered system predicted cancer-specific death and distant metastasis (Table 2). The grade in the three-tiered system remained independent predictors of CSS and DMFS after adjustment for each characteristic. In the univariate setting of non-sarcomatoid ChRCC, the grade in the three-tiered system was still predictors of CSS (Grade 2: HR = 15.4, 95% CI 1.86–128.2; *P* = 0.011) and DMFS (Grade 2: HR = 18.9, 95% CI 2.3–153.6; *P* = 0.006) (Table S1), and the three-tiered grading system remained prognostic indicator for cancer-specific death and distant metastasis after being adjusted each feature (Table S3).

The four-tiered grading system of Avulova et al. [15] is based on a combination of nuclear crowding, atypical cells, and tumor necrosis, which easily results in misclassification. We explored the novel four-tiered grading system, in which the presence of tumor necrosis results in classification as Grade 3. Without tumor necrosis, Grades 1 and 2 are separated on the basis of nuclear crowding and cellular atypia. Grade 4 denotes the presence of sarcomatoid differentiation. Associations between grade of ChRCC in the exploratory four-tiered system with CSS and DMFS according to univariate analysis are listed in Table 2, and Kaplan–Meier curves are shown in Fig. S2. C-indexes of the grading system proposed by Avulova et al. for death from cancer and distant metastasis were 0.892 and 0.897, respectively. The corresponding c-indexes for the exploratory grading system were 0.851 and 0.851. At the same time, we also calculated *c*-indexes (0.875 and 0.877) for the original grading system proposed by Paner et al.

## Discussion

As an external validation dataset, we first validated the studied four-tiered grading system in a cohort of Asian patients with ChRCC. In the present study, we found that grade according to the four-tiered ChRCC grading system proposed by Avulova et al. [15] was significantly associated with cancer-specific death and distant metastasis, and that its predictive ability was stronger than that of any previous grading system. Nevertheless, separating Grades 2 and 3 did not add further prognostic value. Although grade according to the exploratory four-tiered system, which classifies tumors with necrosis as Grade 3, is associated with survival outcomes, its predictive ability is low. Similarly, Grades 2 and 3 have the same predictive power.

It has been shown that Fuhrman tumor grade is unable to predict the prognosis of ChRCC [13, 14, 19]. In the absence of a pathological grading system that accurately predicts the prognosis of ChRCC, several grading systems aimed at identifying patients with adverse outcomes have been proposed. Paner et al.’s system [19], in which 124 patients were studied, and the median follow-up time was 37 months, is reportedly able to accurately stratify patients with ChRCC according to prognosis. This three-tiered ChRCC grading system separates Grades 1 and 2 according to nuclear crowding and atypical cells, whereas Grade 3 tumors have sarcomatoid differentiation. Four recent studies have validated this grading system. Some studies [16–18, 24] have raised questions and dissenting opinions on the three-tiered grade system proposed by Paner et al., Avulova et al.’s study being an exception [25]. In a study of 266 US–American patients, these authors confirmed the prognostic value of chromophobe tumor grade as proposed by Paner et al. for cancer-specific death and distant metastasis [19], which is similar to our results. Cheville et al. studied 124 patients with ChRCC and concluded that, after adjustment for TNM stage, there was no difference in cancer-specific survival between Grades 2 and 1 in Paner et al.’s grading system. In patients with non-sarcomatoid ChRCC, grade in the three-tiered grading system is not associated with cancer-related death rate [16]. The concordance indexes of the studies by Cheville et al. and Avulova et al. for CSS are reportedly 0.766 and 0.84, respectively. According to Finley et al., exclusion of sarcomatoid ChRCC results in grade in the three-tiered grading system no longer being an independent predictor of recurrence [17]. The AUC of the ROC curve for recurrence-free survival in all patients was 0.822. Ohashi et al. reported similar overall survivals as determined by Kaplan–Meier curves for Grades 1 and 2 in the three-tiered chromophobe grading system [18].

In 2019, Ohashi et al. [18] reported a novel two-tiered grading system with a concordance index of 0.79 that was based on findings in 382 patients from multiple medical centers. In this system, the presence of sarcomatoid differentiation and/or necrosis results in a classification of high grade. High grade, advanced age, lymph node and/or distant metastasis, and advanced T stage were associated with overall survival according to multivariate regression analysis. It has been pointed out that overall survival does not reflect cancer-specific survival and that it is inappropriate to include sarcomatoid changes and necrosis in a single grade. Five-year CSS rates reportedly differ significantly between sarcomatoid (44%) and non-sarcomatoid ChRCC (61%) with tumor necrosis [25]. In our study, 5 year CSS rates in the above groups were 37.5% and 90.3%, respectively.

Many studies have found that tumor necrosis is a predictor of prognosis of ChRCC [5, 7, 21, 26, 27]. Avulova et al. [15] accordingly sought to improve Paner et al.’s three-tiered grading system [19] by proposing a four-tiered grading system. As far as we know, this four-tiered grading system has not yet been externally validated. This grading system incorporates necrosis with a high *c*-index of 0.85, which is why we performed external verification on an Asian cohort.

Avulova et al.’s study included 266 patients; the 5 year CSS rate for Grades 1, 2, 3, and 4 was 98.4%, 90.6%, 61.2%, and 44.5%, respectively. They reported that higher grade in their grading system, larger tumor, higher pT stage, pN1 stage, necrosis, and sarcomatoid change were all significantly associated with poor survival rates according to univariate analysis. Additionally, associations between grade according to the four-tiered system and CSS and DMFS outcomes remained significant after adjustment for other variables, which is consistent with our findings. In the subgroup of 247 patients with Grade 1 or 2 tumors, tumor size, pN stage, and necrosis were associated with CSS, whereas pT stage was not. This apparent discrepancy may be attributable to the association between high pT stage and sarcomatoid change. Avulova et al.’s grading system was not evaluated in this subset. In contrast, Ohashi et al. [28] reported that Grade 3 (*P* = 0.871) and Grade 2 (*P* = 0.182) in the four-tiered grading system were not associated with CSS in a cohort of 245 patients.

In our study, LVI was found to be an independent predictor of metastasis after nephrectomy for ChRCC with or without sarcomatoid change. To the best of our knowledge, this is the first such report. Many studies have found that LVI is a predictor of distant metastasis in patients with clear cell renal cell carcinoma [20, 29]. When our analysis was restricted to 67 patients with Grades 2 and 3 in the four-tiered grading system, the risk of death from RCC and metastasis was approximately twice as high for Grade 3 as for Grade 2, and grade tended to be associated with cancer-specific death and metastasis; however, this difference was not statistically significant. Avulova et al. did not analyze or discuss the difference in predictive capability between Grades 2 and 3. We speculate that the association between tumor necrosis and survival may be attributable to the presence of tumor necrosis in sarcomatoid ChRCCs. After adjustment for sarcomatoid differentiation, coagulative tumor necrosis was no longer associated with survival. Despite the c-indexes (0.875 and 0.877) for the three-tiered grading system for CSS and DMFS being lower than those (0.892 and 0.897) for the four-tiered grading system, the increased stratification did not result in more predictors of prognosis. Thus, we consider that the three-tiered grading system more accurately predicts the risk of death and metastasis of ChRCC.

A grading system that includes a separate grade for the presence of tumor necrosis has not previously been explored. In the absence of tumor necrosis, Grades 1 and 2 are separated on the basis of nuclear crowding and cellular atypia. Once tumor necrosis and sarcomatoid differentiation or frank anaplasia have developed, tumors are classified as Grades 3 and 4, respectively. There are statistically significant associations between grade according to this system and CSS and DMFS. However, it has the same problem as the grading system proposed by Avulova et al., in that there is no difference in predictive capability between Grades 2 and 3.

The present study has the following strengths: To the best of our knowledge, this is the first validation of a chromophobe grading system in an Asian cohort. This study was large, being conducted at a large regional medical center. The study patients had all undergone laparoscopic surgery, unlike in the previous studies [15], in which open surgery was performed on most patients. Laparoscopic surgery has fewer complications and is, therefore, currently the preferred surgical approach for most patients. Because this study more closely matches current treatment protocols, the survival times likely more accurately reflect current clinical outcomes. We acknowledge that this study had some limitations. First, it was retrospective and conducted in a single center; a large multicenter study is needed. Like in other studies of ChRCC, the mortality and metastasis rates were low. When there are so few target events, adjustment of multivariable Cox regression analysis by too many variables is prone to result in over-fitting [30]. We, therefore, adjusted only one variable at a time to maximize the reliability of our findings. Nevertheless, our multivariate analysis of multiple variables may not be accurate.

## Conclusions

Although the four-tiered classification grading system proposed by Avulova et al. is of great value in predicting death from ChRCC and metastasis, the risk of death and metastasis was not estimated more accurately for Grade 3 than for Grade 2. Similarly, in another novel exploratory grading system that classifies tumors with necrosis into a separate Grade 3, there was no significant difference in prognostic value between Grades 2 and 3. Therefore, the three-tiered classification grading system proposed by Paner et al. could better define outcomes for patients with ChRCC.

## Supplementary Information

Below is the link to the electronic supplementary material.

Supplementary file1 (DOCX 272 kb)

## Data Availability

The datasets used and/or analyzed during the current study are available from the corresponding author upon reasonable request.
