# Supplementary material for: External validation of a four-tiered grading system for chromophobe renal cell carcinoma
Source: Clin Exp Med. 2024 Mar 30;24(1):61. doi: 10.1007/s10238-024-01314-2 (PMC10981601; doi:10.1007/s10238-024-01314-2)
Supplement: Supplementary file 1 — Supplementary file1 (DOCX 272 kb) [file 10238_2024_1314_MOESM1_ESM.docx]

**Supplementary Data**

**Supplementary Tables**

**Table S1.** Univariable Cox regression analyses of non-sarcomatoid ChRCC for cancer-specific survival

and distant metastasis-free survival.

**Table S2.** Cox regression analyses after being adjusted for each characteristic of all patients for cancer-specific survival and distant metastasis-free survival based on four-tiered ChRCC grade by Avulova et al.

**Table S3.** Cox regression analyses after being adjusted for each characteristic of all patients for cancer-specific survival and distant metastasis-free survival based on three-tiered ChRCC grade by Paner et al.

**Supplementary Figures**

**Figure S1.** Kaplan–Meier curves of cancer-specific survival (A) and distant metastasis-free survival (B) stratified by three-tiered classification grading system by Paner et al.

**Figure S2.** Kaplan–Meier curves of cancer-specific survival (A) and distant metastasis-free survival (B) stratified by exploratory four-tiered classification grading system which classifies tumors with necrosis into a separate Grade 3.

**Table S1.** Univariable Cox regression analyses of non-sarcomatoid ChRCC for cancer-specific survival

and distant metastasis-free survival.

| **Feature** | **CSS** |  | **DMFS** |  |
| --- | --- | --- | --- | --- |
|  | **Univariable** |  | **Univariable** |  |
|  | **HR (95% CI)** | **P** | **HR (95% CI)** | **P** |
| Age (per 10-year increase) | 1.56 (0.83-2.95) | 0.17 | 1.70 (0.93-3.11) | 0.09 |
| Gender (Male VS. Female) | 2.16 (0.48-9.71) | 0.3 | 1.50 (0.37-6.00) | 0.6 |
| Symptom (Yes VS. No) | 1.24 (0.24-4.62) | 0.8 | 1.09 (0.22-5.39) | 0.9 |
| Hypertension (Yes VS. No) | 2.35 (0.52-10.5) | 0.3 | 1.79 (0.43-7.48) | 0.4 |
| DM (Yes VS. No) | 1.32 (0.16-11.0) | 0.8 | 1.23 (0.15-10.0) | 0.8 |
| Charlson score (per score increase) | 2.28 (1.00-5.26) | 0.05 | 2.05 (0.90-4.65) | 0.09 |
| Smoking (Yes VS. No) | 1.5 (0.29-7.72) | 0.6 | 1.23 (0.25-6.12) | 0.8 |
| Alcohol (Yes VS. No) | 2.17 (0.42-11.2) | 0.4 | 1.78 (0.36-8.82) | 0.5 |
| BMI (per 5-kg/m2 increase) | 1.03 (0.33-3.23) | 0.9 | 1.29 (0.45-3.66) | 0.6 |
| Surgery (RN VS. PN) | 2.19 (0.26-18.2) | 0.5 | 2.85 (0.35-23.2) | 0.3 |
| Tumor size (＞7 VS.≤7cm) | 2.06 (0.46-9.20) | 0.3 | 2.89 (0.72-11.6) | 0.13 |
| pT stage (≥T3 VS.≤T2) | 5.79 (1.1-30.2) | 0.04 | 3.93 (0.79-19.6) | 0.09 |
| pN stage (N1 VS. N0) | 158.9 (9.7-2591.7) | ＜0.001 | 76.6 (6.82-859.8) | ＜0.001 |
| LVI (Yes VS. No) | 22.5 (2.7-187.4) | 0.004 | 21.5 (2.63-175.7) | 0.004 |
| Necrosis (Yes VS. No) | 3.69 (0.82-16.6) | 0.09 | 4.83 (1.20-19.4) | 0.03 |
| **Four-tiered ChRCC grade by Avulova et al** | |  |  |  |
| **1** | **1.0 (reference)** |  | **1.0 (reference)** |  |
| **2** | **11.2 (1.17-108.0)** | **0.04** | **11.8 (1.23-113.6)** | **0.03** |
| **3** | **24.7 (2.57-237.4)** | **0.006** | **34.2 (3.83-306.6)** | **0.002** |
| **Three-tiered ChRCC grade by Paner et al** | |  |  |  |
| **1** | **1.0 (reference)** |  | **1.0 (reference)** |  |
| **2** | **15.4 (1.86-128.2)** | **0.011** | **18.9 (2.3-153.6)** | **0.006** |
| Exploratory four-tiered ChRCC grade | |  |  |  |
| 1 | 1.0 (reference) |  | 1.0 (reference) |  |
| 2 | 9.95 (1.03-95.7) | 0.047 | 10.4 (1.08-100.0) | 0.04 |
| 3 | 11.4 (1.18-109.8) | 0.04 | 15.1(1.69-135.7) | 0.02 |
| Abbreviations: CSS Cancer-specific survival; DMFS distant metastasis-free survival; DM Diabetes mellitus; BMI Body mass index; RN Radical nephrectomy; PN Partial nephrectomy; LVI Lymphovascular invasion; HR Hazard ratio; ChRCC Chromophobe renal cell carcinoma | | | | |

**Table S2.** Cox regression analyses after being adjusted for each characteristic of all patients for cancer-specific

survival and distant metastasis-free survival based on four-tiered ChRCC grade by Avulova et al.

| **Feature** | **CSS** |  | **DMFS** |  |
| --- | --- | --- | --- | --- |
|  | **HR (95% CI)** | **P** | **HR (95% CI)** | **P** |
| adjustment for tumor size |  |  |  |  |
| 1 | 1.0 (reference) |  | 1.0 (reference) |  |
| 2 | 11.6 (1.21-111.8) | 0.034 | 12.1 (1.26-116.5) | 0.03 |
| 3 | 22.6 (2.28-224.6) | 0.008 | 30.1 (3.29-275.5) | 0.003 |
| 4 | 226.1 (18.6-2751.3) | <0.001 | 234.0 (21.7-2522.9) | <0.001 |
| adjustment for pT stage |  |  |  |  |
| 1 | 1.0 (reference) |  | 1.0 (reference) |  |
| 2 | 12.0 (1.24-115.4) | 0.032 | 12.1 (1.26-116.9) | 0.03 |
| 3 | 25.7 (2.64-249.8) | 0.005 | 34.1 (3.80-306.5) | 0.002 |
| 4 | 307.2 (29.9-3152.8) | <0.001 | 307.4 (31.8-2967.8) | <0.001 |
| adjustment for pN stage |  |  |  |  |
| 1 | 1.0 (reference) |  | 1.0 (reference) |  |
| 2 | 11.2 (1.17-108.1) | 0.036 | 11.7 (1.22-112.8) | 0.03 |
| 3 | 18.8 (1.85-191.7) | 0.013 | 32.8 (3.65-295.4) | 0.002 |
| 4 | 195.2 (17.8-2143.4) | <0.001 | 272.1 (26.5-2794.8) | <0.001 |
| adjustment for LVI stage |  |  |  |  |
| 1 | 1.0 (reference) |  | 1.0 (reference) |  |
| 2 | 12.2 (1.26-117.7) | 0.031 | 12.9 (1.33-125.1) | 0.03 |
| 3 | 23.4 (2.42-226.7) | 0.006 | 36.7 (4.06-330.6) | 0.001 |
| 4 | 282.8 (28.2-2834.6) | <0.001 | 282.7 (28.2-2828.4) | <0.001 |
| Abbreviations: CSS Cancer-specific survival; DMFS distant metastasis-free survival; LVI Lymphovascular invasion; HR Hazard ratio; ChRCC Chromophobe renal cell carcinoma | | | | |

**Table S3.** Cox regression analyses after being adjusted for each characteristic of all patients for cancer-

specific survival and distant metastasis-free survival based on three-tiered ChRCC grade by Paner et al.

| **Feature** | **CSS** |  | **DMFS** |  |
| --- | --- | --- | --- | --- |
|  | **HR (95% CI)** | **P** | **HR (95% CI)** | **P** |
| adjustment for tumor size |  |  |  |  |
| 1 | 1.0 (reference) |  | 1.0 (reference) |  |
| 2 | 15.2 (1.82-126.6) | 0.012 | 18.2 (2.23-148.2) | 0.007 |
| 3 | 204.4 (17.4-2400.4) | <0.001 | 207.4 (19.8-2170.5) | <0.001 |
| adjustment for pT stage |  |  |  |  |
| 1 | 1.0 (reference) |  | 1.0 (reference) |  |
| 2 | 16.3 (1.95-135.5) | 0.01 | 19.2 (2.36-156.2) | 0.006 |
| 3 | 304.4 (29.6-3126.7) | <0.001 | 297.8 (30.7-2885.3) | <0.001 |
| adjustment for pN stage |  |  |  |  |
| 1 | 1.0 (reference) |  | 1.0 (reference) |  |
| 2 | 13.8 (1.64-115.9) | 0.016 | 18.5 (2.27-150.4) | 0.006 |
| 3 | 186.3 (16.7-2081.5) | <0.001 | 257.0 (24.4-2706.8) | <0.001 |
| adjustment for LVI stage |  |  |  |  |
| 1 | 1.0 (reference) |  | 1.0 (reference) |  |
| 2 | 16.1 (1.93-133.8) | 0.01 | 20.6 (2.50-168.9) | 0.005 |
| 3 | 282.6 (28.0-2850.9) | <0.001 | 272.6 (26.9-2761.1) | <0.001 |
| adjustment for tumor necrosis |  |  |  |  |
| 1 | 1.0 (reference) |  | 1.0 (reference) |  |
| 2 | 13.5 (1.57-117.0) | 0.018 | 15.2 (1.80-128.2) | 0.01 |
| 3 | 176.0 (13.3-2332.4) | <0.001 | 176.2 (15.2-2047.1) | <0.001 |
| Abbreviations: CSS Cancer-specific survival; DMFS distant metastasis-free survival; LVI Lymphovascular invasion; HR Hazard ratio; ChRCC Chromophobe renal cell carcinoma | | | | |


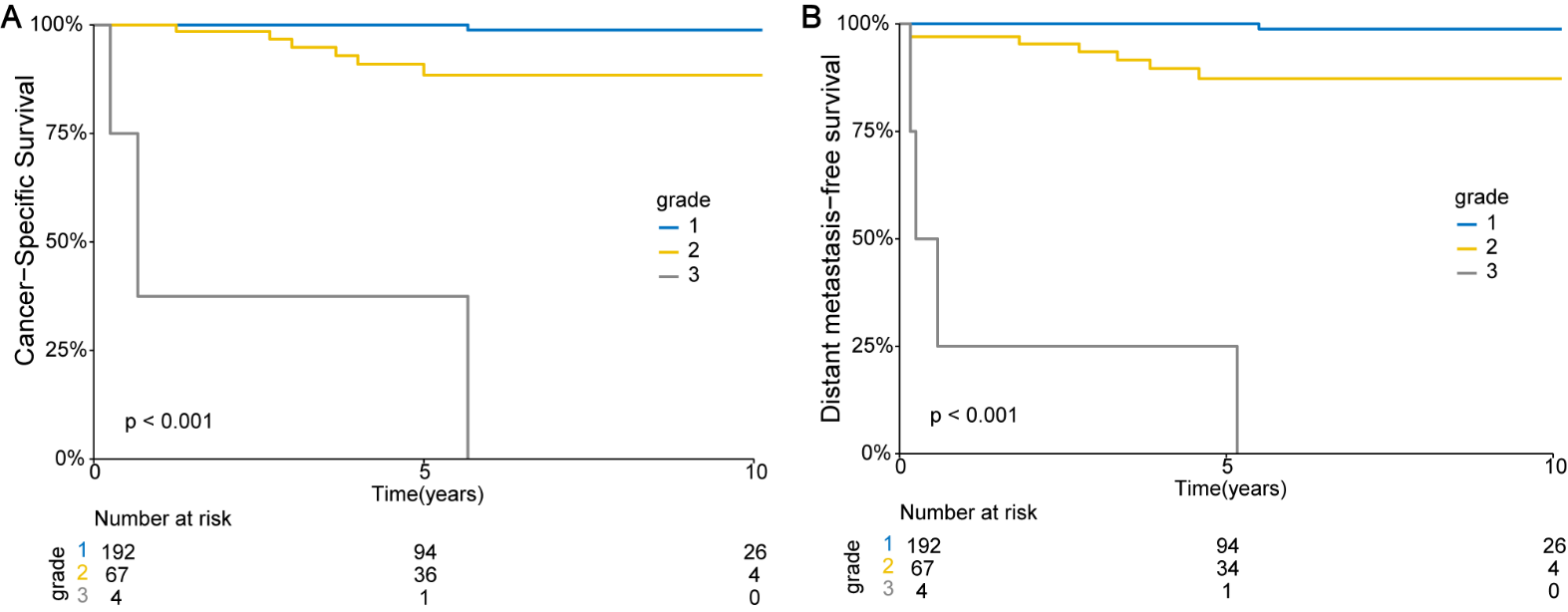


**Figure S1.** Kaplan–Meier curves of cancer-specific survival (A) and distant metastasis-free survival (B) stratified by three-tiered classification grading system by Paner et al.


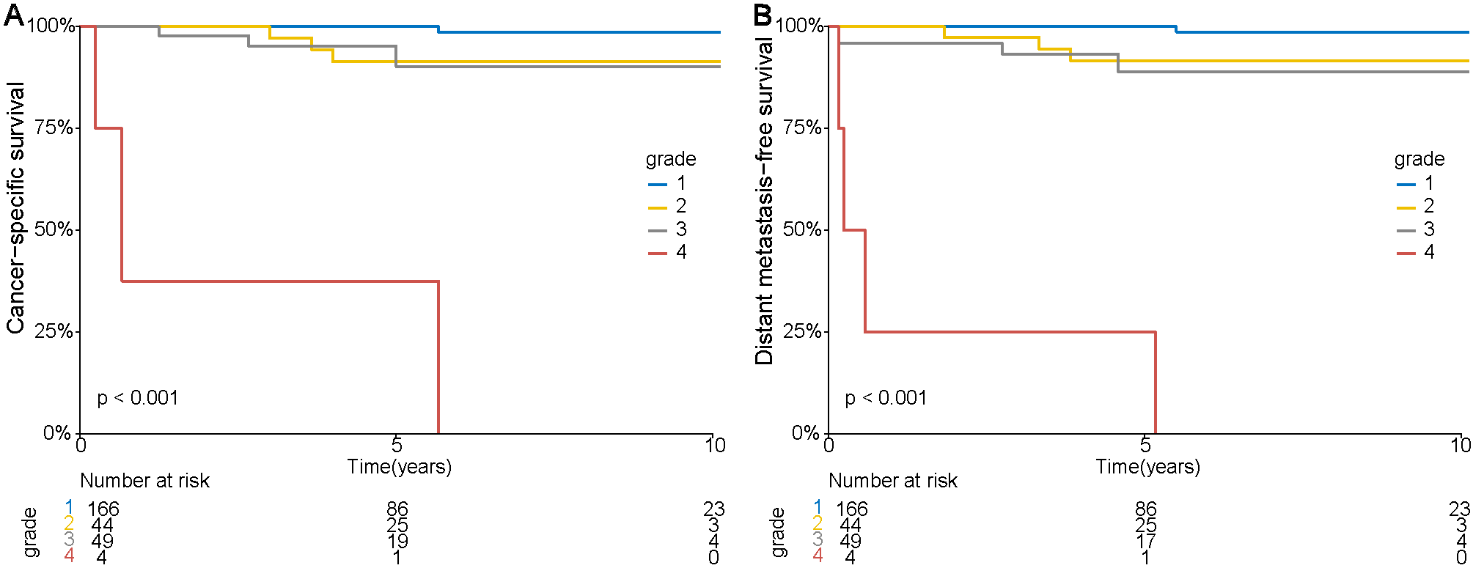


**Figure S2.** Kaplan–Meier curves of cancer-specific survival (A) and distant metastasis-free survival (B) stratified by exploratory four-tiered classification grading system which classifies tumors with necrosis into a separate Grade 3.
